# Normal Laboratory Reference Intervals among Healthy Adults Screened for a HIV Pre-Exposure Prophylaxis Clinical Trial in Botswana

**DOI:** 10.1371/journal.pone.0093034

**Published:** 2014-04-08

**Authors:** Tebogo M. Segolodi, Faith L. Henderson, Charles E. Rose, Kyle T. Turner, Clement Zeh, Peter N. Fonjungo, Richard Niska, Clyde Hart, Lynn A. Paxton

**Affiliations:** 1 United States Centers for Disease Control and Prevention Botswana (CDC Botswana), Gaborone, Botswana; 2 United States Centers for Disease Control and Prevention Atlanta, Atlanta, Georgia, United States of America; 3 United States Centers for Disease Control and Prevention Kenya (CDC-Kenya), Kisumu, Kenya; 4 ICF International, Atlanta, Georgia, United States of America; University of Cape Town, South Africa

## Abstract

**Introduction:**

Accurate clinical laboratory reference values derived from a local or regional population base are required to correctly interpret laboratory results. In Botswana, most reference intervals used to date are not standardized across clinical laboratories and are based on values derived from populations in the United States or Western Europe.

**Methods:**

We measured 14 hematologic and biochemical parameters of healthy young adults screened for participation in the Botswana HIV Pre-exposure Prophylaxis Study using tenofovir disoproxil fumarate and emtricitabine (TDF/FTC) (TDF2 Study). Reference intervals were calculated using standard methods, stratified by gender, and compared with the site-derived reference values used for the TDF2 study (BOTUSA ranges), the Division of AIDS (DAIDS) Grading Table for Adverse Events, the Botswana public health laboratories, and other regional references.

**Results:**

Out of 2533 screened participants, 1786 met eligibility criteria for participation in study and were included in the analysis. Our reference values were comparable to those of the Botswana public health system except for amylase, blood urea nitrogen (BUN), phosphate, total and direct bilirubin. Compared to our reference values, BOTUSA reference ranges would have classified participants as out of range for some analytes, with amylase (50.8%) and creatinine (32.0%) producing the highest out of range values. Applying the DAIDS toxicity grading system to the values would have resulted in 45 and 18 participants as having severe or life threatening values for amylase and hemoglobin, respectively.

**Conclusion:**

Our reference values illustrate the differences in hematological and biochemical analyte ranges between African and Western populations. Thus, the use of western-derived reference laboratory values to screen a group of Batswana adults resulted in many healthy people being classified as having out-of-range blood analytes. The need to establish accurate local or regional reference values is apparent and we hope our results can be used to that end in Botswana.

## Introduction

Clinical laboratory testing is the most widely used medical decision-making tool [1] and is crucial for disease screening, diagnosis, monitoring disease progression and treatment efficacy. Correct interpretation of laboratory tests requires accurate reference intervals from an appropriate population [2]. Reference intervals are typically established by assaying specimens from a sample group of people who meet carefully defined criteria [3]. The reference interval is usually defined as the values encompassing the central 95% of specimens; equating to 2 standard deviations on either side of the mean [4]. Producing reference intervals for a general population is a major challenge, as it requires selecting the appropriate reference population and recruiting individuals who represent relevant demographic groups that meet the inclusion criteria; collecting, processing and testing specimens; and finally, calculating reference values with possible stratification of the data into subgroups.

The currently used reference values in Botswana vary by individual clinical laboratory. For example, some laboratories use values supplied in a manufacturer's kit insert but those values are derived from populations outside the country and may not accurately represent normal values for the Botswana population. Without Botswana-based reference values, there could be considerable misclassification of normal and abnormal test results. This is especially important when implementing lifesaving anti-retroviral (ARV) drug therapy for HIV infected persons. ARVs can have adverse effects which must be recognized early on using appropriate laboratory reference values. As the number of HIV prevention and treatment clinical trials in Botswana has increased greatly, accurate reference values are needed to correctly screen volunteers for study eligibility and as well as monitoring for possible adverse events. A few studies reporting immuno-hematological reference values for the Botswana population exist [5], [6] but there is little, if any, local reference information available for clinical biochemistry parameters in Batswana adults.

Our manuscript reports several hematologic and chemistry parameters among a group of healthy Batswana adults. We used those results to create reference intervals and compared our reference intervals to those already in use in Botswana and in neighboring countries.

## Methods

### The Botswana TDF2 HIV Pre-Exposure Prophylaxis Study (the TDF2 Study)

The laboratory values reported here are from volunteers screened for the TDF2 Study [7]. In brief, this study determined the safety and efficacy of daily oral dosing of the combination antiretroviral drug TDF/FTC (tenofovir disoproxil fumarate/emtricitabine) for the prevention of HIV infection in heterosexually active young adults in Botswana. The study was conducted between March 2007 and May 2010 in the Botswana cities of Gaborone and Francistown. The study enrolled 1219 participants from a total screened population of 2533 sexually active participants aged between 18 and 39 years (median age 24) and randomized them 1∶1 to once daily TDF/FTC or to placebo. Pregnant or breastfeeding participants were excluded from the study and participants who were found to be out of range while screening for the TDF2 were referred to local health settings for assistance while those found to be abnormal after enrollment were taken off study medication prior to being referred to local health settings.

This study is registered with clinicaltrial.gov number NCT00448669.

### Ethical Approval

The study protocol was approved by the CDC Institutional Review Board and the Botswana Health Research Development Committee. Each participant provided written informed consent prior to study screening and enrollment. Parental or guardian assent was also obtained for participants 18–20 years of age considered to be minors as per the Botswana legal system.

### Reference Range Analysis

**Figure 1:**
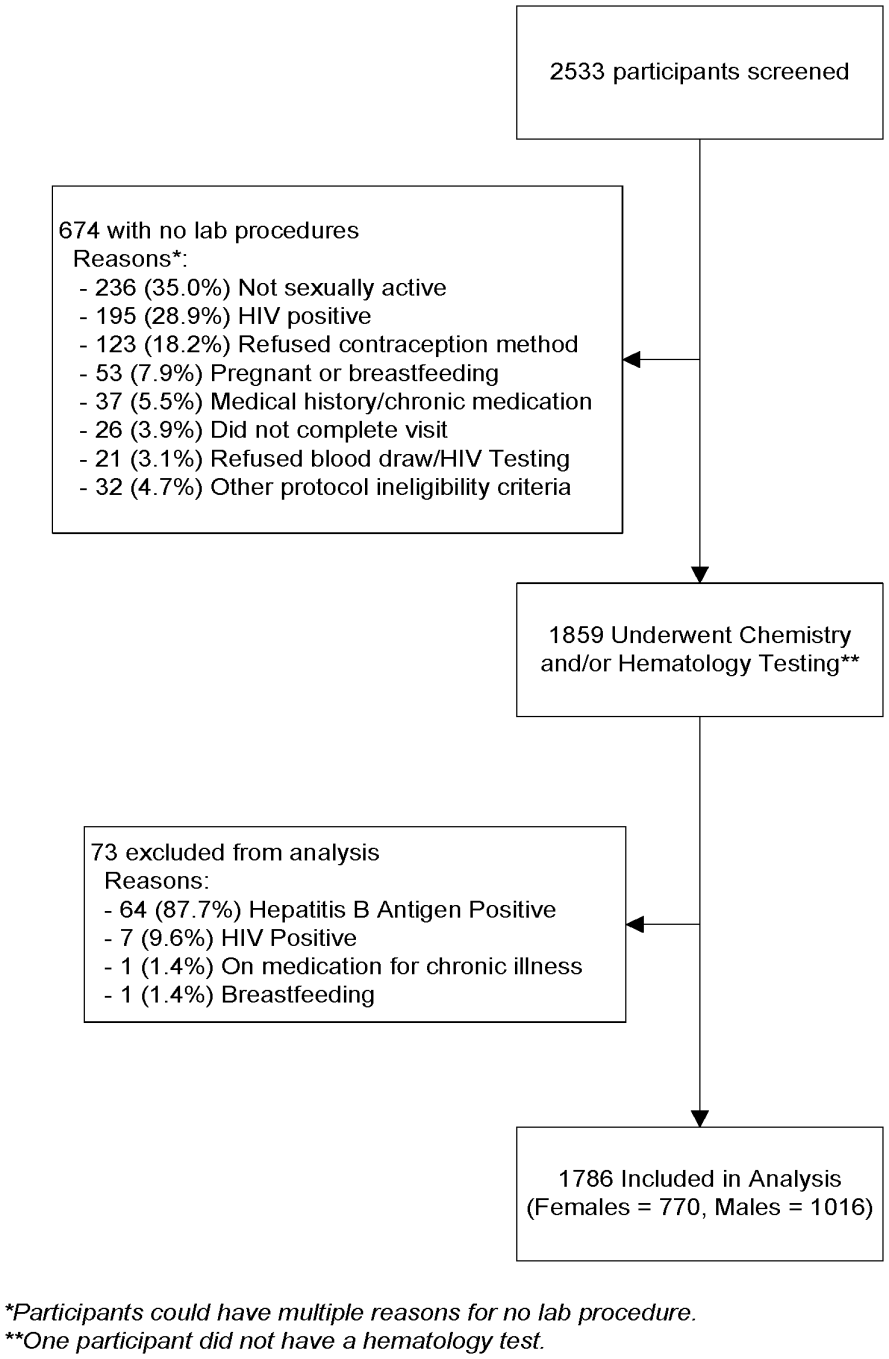
The TDF2 Botswana PrEp lab reference values Consort diagram.

Of the 1859 TDF2 study participants who underwent chemistry and hematology testing, 73 were excluded from analysis due to factors that rendered them ineligible for study participation and could have affected their suitability for reference values. Of the 73 excluded, 64 (87.7%) were positive for HBsAg, 7 (9.6%) were HIV-positive, 1 (1.4%) was on medication for chronic illness and 1 (1.4%) was breastfeeding. This left a total sample of 1786 participants from whom the reference ranges were calculated (Figure 1). The chemistry and hematology tests performed included hemoglobin (Hb) and hematocrit (Hct), creatinine, inorganic phosphorus, bicarbonate (HCO_3_), potassium, sodium, chloride, blood urea nitrogen (BUN), direct and total bilirubin, serum amylase, aspartate amino transferase (AST) and alanine aminotransferase (ALT).

The TDF2 study used two reference range tables to classify laboratory abnormalities: the BOTUSA site derived reference ranges (Table 1 column 3) were used to classify results as normal or abnormal and together with the study exclusion/inclusion criteria used to decide on study participation of a client. Grading values derived from the 2004 Division of AIDS (DAIDS) grading system (Table 1 columns 5–8) were used for grading adverse events during the trial. The BOTUSA site-derived reference ranges were extracted from what the Botswana public health laboratories used at the inception of the TDF2 study and derived from western populations according to the methodologies existent then. Both the DAIDS and the TDF2 study reference ranges were derived from western populations.

**Table 1:** The comparison of calculated population biochemical reference values for participants screened (N = 1786; Females = 770 and Males = 1016) for the TDF2 Botswana study with DAIDS and BOTUSA site established ranges.

Analyte	Units	Comparison (out of range)		Division of AIDS Toxicity Grading (DAIDS)			
		BOTUSA Reference Interval	TDF2	Grade 1	Grade 2	Grade 3	Grade 4
			N (%)	N (%)	N (%)	N (%)	N (%)
**AST (SGOT)**	IU/L	10–36	71 (3.98)	29 (1.62)	5 (0.28)	1 (0.06)	0 (0.00)
**ALT (SGPT)**	IU/L	11–41	401 (22.45)	33 (1.85)	2 (0.11)	0 (0.00)	0 (0.00)
**Creatinine**	mg/dL	0.7–1.4	572 (32.03)	0 (0.00)	0 (0.00)	0 (0.00)	0 (0.00)
**Blood Urea Nitrogen**	mg/dL	7.0–30.0	232 (12.99)	1 (0.06)	0 (0.00)	0 (0.00)	0 (0.00)
**Bilirubin (Total)**	mg/dL	0.1–1.3	110 (6.16)	82 (4.59)	26 (1.46)	2 (0.11)	0 (0.00)
**Bilirubin (Direct)**	mg/dL	0.1–0.4	69 (3.86)	25 (1.40)	16 (0.90)	0 (0.00)	0 (0.00)
**Amylase**	IU/L	60–97	908 (50.84)	492 (27.55)	182 (10.19)	44 (2.46)	1(0.06)
**Phosphate (inorganic)**	mg/dL	2.5–4.5	150 (8.40)	39 (2.18)	106 (5.94)	26 (1.46)	0 (0.00)
**Chloride**	mEq/L	95–108	14 (0.78)	14 (0.78)	0 (0.00)	0 (0.00)	0 (0.00)
**CO_2_**	mEq/L	21–29	146 (8.17)	94 (5.26)	0 (0.00)	1 (0.06)	0 (0.00)
**Potassium (Indirect)**	mEq/L	3.5–5.1	70 (3.92)	26 (1.46)	1 (0.06)	0 (0.00)	0 (0.00)
**Sodium (Indirect)**	mEq/L	135–145	24 (1.34)	22 (1.23)	1 (0.06)	1 (0.06)	0 (0.00)
**Hemoglobin Males**	g/dl	13–17	110 (6.16)	35 (1.96)	15 (0.84)	16 (0.90)	2 (0.11)
**Hemoglobin Females**	g/dl	12–15	193 (10.81)				
**Hematocrit Males**	%	40–50	125 (7.00)	-	-	-	-
**Hematocrit Females**	%	36–45	147 (8.23)				

### Blood Collection and Testing Methodologies

Blood specimens were collected in the clinics at each study site and transported in coolers from the on-site clinic laboratories to the off-site laboratories which were a 5 to10 minute drive away. For hematology, the blood was collected in a Becton Dickinson (BD) ethylene diamine tetra-acetic acid (EDTA) Vacutainer tube (Franklin Lakes, NJ, USA), while that for biochemistry was collected in a BD serum separation tube (SST) that was centrifuged (3,000× g, 3 minutes) within two hours of sample collection.

Prior to use, both chemistry and hematology instruments ware validated on site using validation panels from Contract Laboratory Services (CLS) South Africa. A Sysmex XT1800i hematology analyzer (Symex, Kobe, Japan) was used for hemoglobin and hematocrit within 24 hours of whole blood sample collection as recommended by the manufacturer and a Roche Integra 400plus analyzer was used for chemistry tests. To maintain internal quality control, the equipment had to satisfy the calibration criteria prior to analysis, only reagents within expiration dates were used, and a second laboratory technologist had to validate the results of the first reader and authorize them for release. Testing staff were Good Clinical Laboratory Practices (GCLP) certified and had to be audited against the GCLP standards quarterly. For hematology testing each day, at least two levels of e-check controls (levels 1, 2, or 3) were run and both results had to be within range before testing patient samples; for chemistry tests, control results for two level of controls s (pathological/abnormal and non-pathological/normal) had to be shown to be within acceptable ranges before testing participants' samples. Proficiency testing panels were performed three times a year and evaluated by the College of American Pathologists as the external quality control.

### Statistical Analysis

The Clinical Laboratory Standards Institute (CLSI) [8] recommends a sample size of at least 120 values for non-parametric reference intervals and for parameters not influenced by gender (60 females and 60 males). For parameters that are influenced by gender, the minimum recommended number is 240 reference individuals (120 females and 120 males). Hence, our study sample size (1786; 770 females and 1016 males) exceeded the recommended CLSI (formerly NCCLS) guideline.

Reference values were estimated using non-parametric methods. The mean (with 95% confidence interval), median, range, and 2.5 and 97.5 percentiles were computed for each analyte for the total sample and by gender. Means and the corresponding 95% CI were computed using bootstrapping. We created 1,000 datasets by randomly selecting the participants, with replacement, and then summarizing to obtain the mean and 95% CI. A non-parametric Wilcoxon rank-sum test was performed to test for gender differences for each analyte. The reference interval, according to CLSI, is defined as the interval between and including the upper and lower reference limits, which are estimated to enclose a specified percentage (here 95%) of the values for a population from which the reference subjects were drawn. Our calculated analyte values were compared to the DAIDS reference intervals. The overall percentage outside the specified interval, as well as the percentage of analyte values in each of the severity grades (1–4), was computed. Lastly, our calculated analyte reference intervals were compared to those of other African countries.

## Results

### Characteristics of Study Population

Of the 1786 screened participants, 1016 (56.9%) were males while 770 (43.1%) were females. 41 of the 1786 participants (2.3%) were 18–20 years old; 1618 (90.6%) were 21–29 years old; and 127 (7.1%) were 30–39 years old. The median age was 24 years (IQR, 4years). There were. About half of the participants were from each site [Gaborone (877; 49.1%) and Francistown (909; 50.9%)] and 1045 (58. 5%) reported alcohol use within the prior three months.

### Hematological and Biochemistry Parameters

The analysis results are presented in Table 2. We observed statistically significant differences in hemoglobin and hematocrit by gender, with males having higher values than females. Similarly, males had significantly higher values than females for most biochemistry parameters, including CO2, potassium, AST, ALT, creatinine, blood urea nitrogen, total bilirubin, direct bilirubin and amylase. However, females had significantly higher values than males for chloride and phosphate parameters.

**Table 2:** The calculated population biochemical reference values for participants screened (N = 1786; Females = 770 and Males = 1016) for the TDF2 Botswana study.

Analyte	Sex	Mean	95% CI for mean	Median	Range	2.5^th^–97.5^th^ percentile	p-value*
**Aspartate Aminotransferase**	Combined	21.55	(21.09, 22.04)	20.0	10.0–204.0	13.0–42.0	<0.001
**(AST/SGOT)**	Female	18.57	(18.13, 19.14)	17.0	10.0–134.0	12.0–31.0	
**IU/L**	Male	23.80	(23.12, 24.61)	22.0	11.0–204.0	14.0–48.0	
**Alanine Aminotransferase**	Combined	17.48	(16.96, 18.02)	15.0	0.0–152.0	7.0–46.0	<0.001
**(ALT/SGPT)**	Female	14.25	(13.64, 14.87)	12.0	0.0–148.0	7.0–33.0	
**IU/L**	Male	19.94	(19.20, 20.68)	17.0	3.0–152.0	8.0–53.0	
**Creatinine**	Combined	0.74	(0.73, 0.74)	0.7	0.3–1.4	0.5–1.1	<0.001
**mg/dL**	Female	0.62	(0.61, 0.62)	0.6	0.3–1.1	0.5–0.8	
	Male	0.83	(0.82, 0.83)	0.8	0.5–1.4	0.6–1.1	
**Blood Urea Nitrogen**	Combined	10.19	(10.00, 10.38)	9.0	3.0–75.0	5.0–21.0	<0.001
**mg/dL**	Female	9.72	(9.43, 10.00)	9.0	3.0–37.0	5.0–21.0	
	Male	10.54	(10.28, 10.82)	10.0	4.0–75.0	5.0–22.0	
**Bilirubin (Total)**	Combined	0.66	(0.64, 0.68)	0.5	0.1–3.6	0.2–1.8	<0.001
**mg/dL**	Female	0.51	(0.48, 0.53)	0.4	0.1–3.0	0.2–1.3	
	Male	0.78	(0.75, 0.81)	0.7	0.2–3.6	0.3–2.1	
**Bilirubin (Direct)**	Combined	0.18	(0.17, 0.18)	0.2	0.0–0.8	0.1–0.4	<0.001
**mg/dL**	Female	0.14	(0.14, 0.15)	0.1	0.0–0.8	0.0–0.3	
	Male	0.21	(0.20, 0.21)	0.2	0.0–0.8	0.1–0.5	
**Amylase**	Combined	96.11	(94.59, 97.82)	91.0	27.0–473.0	47.0–176.0	<0.001
**IU/L**	Female	90.99	(88.91, 93.38)	87.0	32.0–417.0	46.0–162.0	
	Male	99.99	(97.61, 102.35)	94.0	27.0–473.0	49.0–181.0	
**Phosphate (Inorganic)**	Combined	3.27	(3.24, 3.29)	3.3	1.3–5.2	2.2–4.3	<0.001
**mg/dL**	Female	3.37	(3.34, 3.41)	3.4	1.8–5.0	2.3–4.4	
	Male	3.18	(3.15, 3.22)	3.2	1.3–5.2	2.0–4.3	
**Chloride**	Combined	102.61	(102.50, 102.72)	103.0	95.0–112.0	98.0–107.0	<0.001
**mEq/L**	Female	103.63	(103.49, 103.79)	103.0	95.0–112.0	100.0–108.0	
	Male	101.84	(101.70, 101.97)	102.0	96.0–112.0	98.0–106.0	
**CO_2_**	Combined	24.71	(24.60, 24.82)	24.7	10.0–32.8	19.9–29.1	<0.001
**mEq/L**	Female	23.57	(23.43, 23.71)	23.5	16.9–30.4	19.2–27.7	
	Male	25.58	(25.45, 25.71)	25.6	10.0–32.8	21.3–29.5	
**Potassium (Indirect)**	Combined	4.32	(4.30, 4.34)	4.3	2.9–5.8	3.6–5.2	0.006
**mEq/L**	Female	4.28	(4.26, 4.31)	4.3	2.9–5.8	3.6–5.1	
	Male	4.34	(4.32, 4.37)	4.3	3.1–5.7	3.6–5.2	
**Sodium (Indirect)**	Combined	139.08	(138.98, 139.18)	139.0	127.0–156.0	135.0–143.0	<0.001
**mEq/L**	Female	138.86	(138.72, 139.01)	139.0	132.0–146.0	135.0–143.0	
	Male	139.24	(139.11, 139.37)	139.0	127.0–156.0	135.0–143.0	
**Hemoglobin (Hg)**	Combined	14.46	(14.36, 14.54)	14.7	6.4–24.9	10.4–17.6	<0.001
**g/dL**	Female	12.87	(12.76, 12.97)	13.0	6.4–16.6	9.1–15.3	
	Male	15.66	(15.59, 15.74)	15.6	9.4–24.9	13.2–17.8	
**Hematocrit (Hct)**	Combined	43.13	(42.90, 43.33)	43.5	23.8–68.0	32.9–51.7	<0.001
**%**	Female	39.17	(38.90, 39.41)	39.4	23.8–50.2	30.6–45.6	
	Male	46.12	(45.93, 46.31)	46.0	35.0–68.0	40.2–52.9	

Note:

*P-value is for the Wilcoxon rank-sum test for Females versus Males.

Using the western-derived BOTUSA reference intervals results, over 50% of our healthy volunteers were out-of-range for amylase (Table 1) and over 10% were out of range for ALT, creatinine, BUN, and hemoglobin (for females). Using DAIDS grading system, established using the BOTUSA site reference values 40.3% of our participants had abnormal amylase levels and at least 45 participants would have had a severe or life threatening (Grade 3) amylase abnormality (Table 2). Furthermore, 18 and 26 participants would have been classified as having severe or life threatening hemoglobin and inorganic phosphate levels, respectively.

We found noticeable differences between the newly established reference intervals by this study and those used in other settings. Compared to other intervals currently in use in Botswana, our reference intervals had higher upper limits for ALT, BUN, bilirubin (total), bilirubin (direct) amylase and phosphate, depressed lower limits for ALT, creatinine, BUN, hemoglobin and hematocrit (women only), but comparable levels for chloride, potassium, sodium and hematocrit (men only) (Table 3). Compared to regional-derived values from the Combined Eastern and Southern Africa and Combined Uganda, Kenya and Zambia reports, we had lower AST, ALT, creatinine, total and direct bilirubin values yet higher upper limit intervals for AST and ALT compared to those from the US Massachusetts General Hospital (Table 3). The upper limit of our amylase interval was comparable to that from the US Massachusetts General Hospital (MGH) but significantly higher than that from the Combined Eastern and Southern Africa report (Table 3). The hematological values were comparable to the combined regional intervals and the MGH values, particularly in males. For females, our lower limit fell outside the MGH intervals and those currently in use in Botswana.

**Table 3:** Comparison of the calculated Botswana TDF2 screened cohort reference intervals with the reference intervals used in Botswana and other African countries.

Analyte	Units	Botswana TDF2 Screened Cohort	Botswana Ministry of Health (MOH)*	Botswana Ministry of Health (MOH)**	Published Botswana Harvard Partnership Lab	US Massachusetts General Hospital	Combined Eastern and Southern African	Combined Study (Uganda, Kenya, Zambia)
**AST (SGOT)**	IU/L	13–42	10–34	11–41	-	0–35	14–60	14–60
**ALT (SGPT)**	IU/L	7–46	11–41	10–34	-	0–35	8–61	8–61
**Serum Creatinine**	mg/dL	44.2–97.2	53–100	53–97	-	0–133	47–109	47–109
**Blood Urea Nitrogen**	mg/dL	5.0–21.0	5.6–19.7	5.6–19.7	-	10–20	-	-
**Bilirubin (Total)**	mg/dL	0.2–1.8	0.1–1.5	0.1–1.5	-	0.3–1.0	0.2–2.2	-
**Bilirubin (Direct)**	mg/dL	0.1–0.4	0–0.2.	0–0.2	-	0.1–0.3	0–0.5	-
**Amylase**	IU/L	47–176	0–108	28–100	-	60–180	35–159	-
**Phosphate (Inorganic)**	mg/dL	2.2–4.3	1.91–2.35	0.80–1.55	-	-	-	-
**Chloride**	mEq/L	98–107	95–108	95–108	-	-	-	-
**CO_2_**	mEq/L	19.9–29.1	-	-	-	-	-	-
**Potassium (Indirect)**	mEq/L	3.6–5.2	3.5–5.1	3.5–5.1	-	-	-	-
**Sodium (Indirect)**	mEq/L	135–143	135–145	135–145	-	-	-	-
**Hemoglobin**	g/dl							
**Men**		13.2–17.8	13.7–18.0	13.7–18.0	11.90–17.10	13.5–17.5	12.2–17.7	12.2–17.0
**Women**		9.1–15.3	12.0–16.0	12.0–16.0	9.3–16.00	12–16	9.5–15.8	9.5–15.8
**Hematocrit**	%							
**Men**		40.2–52.9	40–54	40–54	36.10–49.30	41–53	35.0–50.8	35.0–50.8
**Women**		30.6–45.6	36–48	36–48	28.2–46.2	36–46	29.4–45.4	29.4–45.4

*Botswana Ministry of health Patient management system Roche Integra derived values.

**Botswana Ministry of health reference values using the Beckman coulter AU680 analyzer.

## Discussion

There is paucity of reference interval data in Botswana with only a few studies conducted on hematological/immunohematological ranges and one on lipids [5]–[6], [9] for the biochemical ranges. Reference intervals for laboratory parameters provide important data for assessment of the health status of an individual. For this reason reference intervals are routinely used in clinical trials at enrollment to determine eligibility, establish baseline measures, and to monitor participants' health during the course of the trial. Moreover, analytes including hemoglobin, bilirubin and neutrophils are used as markers for the presence of disease. Without locally derived reference values for African populations, clinicians and researchers often must use reference values obtained from European or North American populations. However, several African studies have highlighted differences between locally derived values and western derived values. Hence, over the last decade, there has been an increase in the number of studies aimed at establishing locally derived reference intervals for more effective patient management and proper conduct of clinical research in these settings. The use of population based ranges for the TDF study (Table 1) to assess laboratory data, would have led to a reduction in out of range clinical trial volunteers for Amylase, BUN, ALT hemoglobin, inorganic phosphate which could have translated to rapid enrollment of participants and a savings on resources utilized for attending to adverse events. Using population based reference ranges to work out toxicity tables for the study would also have resulted in fewer participants classified as grades 1–4 and requiring management and intensified monitoring.

Similar to other African studies, a substantial number of our TDF2 participants would have been excluded from participating in a clinical trial if the instrument/assay kit-derived values currently in use in most hospitals were applied (Table 3). A study in Uganda reported up to 31% exclusion using western-derived intervals for recruitment into a clinical trial while only 17% of these participants would have been excluded if they had used locally established reference intervals [10]. Similarly, a Kenya study reported up to a 40% exclusion rate if western-derived values were applied compared to population based reference intervals for a rural population [11]. These misclassifications have a negative impact by wasting scarce resources and creating time delays in clinical trials and more importantly in creating misdiagnoses. Therefore, the need to have accurate, locally-derived reference intervals is a fundamental requisite for establishing adequate medical care and conducting more efficiently run clinical trials for the benefit of African populations.

Significant gender differences for hemoglobin and hematocrit levels in our study are consistent with previous reports of higher Hb and Hct levels in males than females and can be explained by the likely effect of androgens on erythropoiesis that increases the number of circulating RBC's with a resultant hemodilution [11]–[15]. The lower Hb levels in women are a common phenomenon in several other African studies [10]–[12], [15]–[19] these levels can further be exacerbated by poor nutritional status resulting in iron deficiency, genetic disorders including thalassemia and sickle cell trait or infections with helminthes, malaria or schistosomiasis for which we did not test. Our findings that males have higher levels of CO2, potassium, AST, ALT, creatinine, blood urea nitrogen, bilirubin (total and direct), and amylase, and lower levels of chloride and phosphate (inorganic) is consistent with previous studies [10]–[11], [15]. Even though we observed significant differences in a majority of parameters, these may not be of medical significance and warrants further investigation.

Several limitations could be cited for our study. Though the study population was from the cities of Francistown and Gaborone with representative populations from most of the areas of Botswana, the ranges established cannot be generalized to the whole of Botswana. Information on other factors that could possibly affect analyte levels such as diet, lifestyle were not collected from the participants. Additionally, although alcohol may affect several biochemical parameters including Amylase, this was not investigated in the present study. The inclusion of women on hormonal contraceptives in this study population means that hormonal effects on biochemical and hematological analytes could not be ruled out. While the study used a single platform for biochemistry and hematology, the CLSI guidelines require that individual laboratories perform evaluations with limited sample size to verify the applicability of such references within their setting. Moreover the platforms used in this study are commonly used in the Botswana public health setting.

Even with the above limitations, our results were still comparable with other studies in the region. Our hemoglobin and hematocrit intervals were comparable to those obtained in a recent Botswana study, [6] but there were variations that were likely due to a larger geographic area and a more ethnically diverse population in our sample. In addition, our intervals came from a larger sample size and they agreed overall with the recently published combined Eastern and Southern Africa consensus intervals, [19] except serum amylase. However, our amylase intervals were similar to the intervals in a study of health Ugandan blood donors [10] indicating that these levels are not uncommon in the African setting. Though the gender differences for some parameters in our study are statistically significant, the absolute value of those differences is relatively small. It may be that these differences are not of sufficient clinical significance to warrant the establishment of different standards between men and women. The accumulation of more reference data from other sources will help to clarify that question.

Given that the frequency of clinical trials and persons receiving clinical services is increasing substantially in sub-Saharan Africa, the introduction of geographic and ethnically valid laboratory intervals is needed for establishing accurate toxicity tables for use in patient management as well as in recruiting and monitoring participants in clinical trials in addition to the DAIDS toxicity grading tables. The established intervals are appropriate for use in young adult population in Botswana and need to be validated using a smaller sample size as indicated in the CLSI guidelines [8]. The accuracy of our tables will increase as more data from representative populations becomes available. As this process of revising the tables unfolds, it will be important to verify that the revised intervals do in fact represent healthy norms for Africa. For that reason, it is important that interventional trials should continue to carefully review their data to ensure that persons whose laboratory values fall within the new ranges are indeed at no greater risk as compared to the current tables. Our reference intervals, in addition to the existing intervals could be used for comparison by the Botswana Ministry of health in establishing Botswana population based reference ranges for standardization of laboratory diagnosis.
